# Draft Genome Sequence of Streptococcus agalactiae UMB7782, Isolated from the Female Urinary Tract

**DOI:** 10.1128/MRA.00423-20

**Published:** 2020-05-14

**Authors:** Linda M. Salgado, Taylor Miller-Ensminger, Adelina Voukadinova, Alan J. Wolfe, Catherine Putonti

**Affiliations:** a Department of Biology, Loyola University Chicago, Chicago, Illinois, USA; b Bioinformatics Program, Loyola University Chicago, Chicago, Illinois, USA; c Department of Microbiology and Immunology, Stritch School of Medicine, Loyola University Chicago, Maywood, Illinois, USA; d Department of Computer Science, Loyola University Chicago, Chicago, Illinois, USA; University of Maryland School of Medicine

## Abstract

Streptococcus agalactiae is a Gram-positive bacterium common to the human gut and vaginal microbiota. Here, we report the 2.1-Mbp draft genome sequence of S. agalactiae UMB7782, isolated from a urine sample from a woman with a recurrent urinary tract infection.

## ANNOUNCEMENT

Streptococcus agalactiae, also known as a group B *Streptococcus*, is known as both a commensal bacterium and a pathogen in humans and animals (see reviews [1, 2]). While sometimes pathogenic (2), S. agalactiae is found in 20 to 40% of healthy women, colonizing the urogenital and/or lower gastrointestinal tract (3–6). While a part of the normal urogenital flora of many women, S. agalactiae can cause urinary tract infections (UTIs) (2). The rise of antibiotic-resistant strains of S. agalactiae is a pressing concern for this emerging uropathogen (2, 7). Here, we present the draft genome of S. agalactiae UMB7782, isolated from a voided urine sample obtained from a female with a recurrent UTI.

S. agalactiae UMB7782 was isolated from a previous institutional review board (IRB)-approved study (University of California, San Diego, IRB no. 170077AW) using the expanded quantitative urinary culture (EQUC) protocol (8). The sample was collected from a patient at the Women’s Pelvic Medicine Center at the University of California, San Diego, California, in August 2017. The genus and species of the isolate was determined by matrix-assisted laser desorption ionization–time of flight (MALDI-TOF) mass spectrometry (8) before storage at −80°C. The isolate was streaked onto a Columbia nalidixic acid (CNA) agar plate and incubated at 35°C with 5% CO_2_ for 24 h. A single colony was selected and grown overnight in blood heart infusion (BHI) liquid medium under the same conditions. DNA was extracted using the Qiagen DNeasy blood and tissue kit’s Gram-positive extraction protocol and quantified using a Qubit fluorometer. The extraction protocol was slightly modified; we used 230 μl of lysis buffer (180 μl 20 mM Tris-Cl, 2 mM sodium EDTA, and 1.2% Triton X-100 and 50 μl of lysozyme) in step 2 and reduced the incubation time in step 5 to 10 min. DNA was sent to the Microbial Genome Sequencing Center (MiGS) at the University of Pittsburgh for library preparation and sequencing. There the DNA was first enzymatically fragmented using an Illumina tagmentation enzyme, and indices were attached via PCR. The library was sequenced on the Illumina NextSeq 550 platform, producing 1,612,404 pairs of 150-bp reads. The raw reads were trimmed using Sickle v1.33 (https://github.com/najoshi/sickle). The reads were assembled using SPAdes v3.13.0 with the “only-assembler” option for k values of 55, 77, 99, and 127 (9). Genome coverage was calculated using BBMap v38.47 (https://sourceforge.net/projects/bbmap/). Additionally, the NCBI PGAP v4.11 (10) was used to annotate the sequence. Unless specifically noted, default parameters were used for each software tool.

The S. agalactiae UMB7782 draft genome is 2,136,704 bp long with a GC content of 35.11%. It was assembled into 32 contigs with a genome coverage of 197× and an *N*_50_ score of 147,539 bp. The PGAP annotation found 2,051 protein-coding genes, 47 tRNAs, and 5 rRNAs. The genome assembly also was examined using the ResFinder Web server v3.2 (11). Resistances to macrolides and tetracycline were predicted. Both have previously been reported for other members of the species (2). Further analysis will help us better understand the pathogenic nature of this species in the female urinary tract.

### Data availability.

This whole-genome shotgun project has been deposited in GenBank under the accession no. JAAUVX000000000. The version described in this paper is the first version, JAAUVX010000000. The raw sequencing reads have been deposited in the SRA under the accession no. SRR11441028.
